# Short day length-induced decrease of cesium uptake without altering potassium uptake manner in poplar

**DOI:** 10.1038/srep38360

**Published:** 2016-12-07

**Authors:** Yusaku Noda, Jun Furukawa, Tsutomu Aohara, Naoto Nihei, Atsushi Hirose, Keitaro Tanoi, Tomoko M. Nakanishi, Shinobu Satoh

**Affiliations:** 1 Graduate school of Life and Environmental Sciences, University of Tsukuba, Tsukuba 305-8572, Japan; 2 Faculty of Life and Environmental Sciences, University of Tsukuba, Tsukuba 305-8572, Japan; 3 Center for Research in Isotopes and Environmental Dynamics, University of Tsukuba, Tsukuba 305-8577, Japan; 4 Graduate school of Agricultural and Life Sciences, The University of Tokyo, Tokyo 113-8657, Japan; 5 PRESTO, Japan Science and Technology Agency (JST), Kawaguchi, 332-0012, Japan

## Abstract

Short day length-induced alteration of potassium (K) localization in perennial trees is believed to be a mechanism for surviving and adapting to severe winters. To investigate the relationship between cesium (Cs) and K localizations, a model tree poplar, hybrid aspen T89, was employed. Under short day length conditions, the amount of ^137^Cs absorbed through the root and translocated to the root was drastically reduced, but ^42^K was not. Potassium uptake from the rhizosphere is mediated mainly by KUP/HAK/KT and CNGC transporters. In poplar, however, these genes were constantly expressed under short-day conditions except for a slight increase in the expression a *KUP/HAK/KT* gene six weeks after the onset of the short-day treatment. These results indicated that the suppression of ^137^Cs uptake was triggered by short day length but not regulated by competitive Cs^+^ and K^+^ transport. We hypothesize that there are separately regulated Cs^+^ and K^+^ transport systems in poplar.

In 2011, the Fukushima Daiichi Nuclear Power Plant accident released a large amount of radionuclides into the environment. Due to its long half-life, radioactive cesium (^137^Cs) was considered the main contaminant. To estimate the transfer process of ^137^Cs in the terrestrial environment, we focused on its behavior in woody plants because the transfer process within the forest ecosystem is much slower than it is in other areas^1^. After the forest contamination by ^137^Cs, depositions to tree canopies, leaf- and/or bark uptake, acropetal branch translocation, etc. were energetically investigated^2^,^3^,^4^. Little is known about the physiology of Cs transfer and distribution within trees. Cesium chemically resembles potassium (K) but it is not an essential nutrient for plant growth. Avery reported that Cs^+^ inhibits the inward-K^+^ channels in the plasma membrane and is therefore considered toxic to plants^5^. In general, rhizosphere Cs^+^ consists mostly of stable ^133^Cs and its concentration is <200 μM, which is not toxic to plants^6^. Cesium uptake and translocation within the plant body are thought to be mediated by K^+^ transport systems^6^. *Arabidopsis* HAK5 (AtHAK5) is a K^+^ uptake transporter in roots and is up-regulated under K^+^ deficiency^7^,^8^. The *AtHAK5* T-DNA insertion mutant, *athak5*, showed a significantly decreased K content and a tolerance to 300 μM Cs^+^ treatment under low K conditions^9^,^10^. AtCNGC2 demonstrated cation transport activity using transgenic transfected human embryonic kidney cells. *AtCNGC1* is the candidate gene for Cs^+^ uptake and was identified by quantitative trait locus analysis in *Arabidopsis*^11^,^12^.

In this study, we investigated ^137^Cs and ^42^K localizations using a model tree, poplar, under both long- and short photoperiods. To estimate the ^137^Cs retention time within the forest ecosystem, the Cs content of each tree organ must be determined under seasonal conditions since Cs is circulated via root uptake, translocation, and leaf abscission. Poplar is a perennial deciduous tree with a characteristic seasonal cycle of growth and dormancy. The phase shift from growth to dormancy is a winter adaptation^13^. The transition of meristems into dormant buds is crucial to protect them against hazardous frosts. Woody plants shift their growth stage when they perceive changes in photoperiod and temperature^14^. The initiation of cold acclimation under short day length increases endogenous abscisic acid levels and reduces endogenous gibberellic acid levels^15^,^16^,^17^. In beech tree (*Fagus sylvatica* L.) leaf senescence, leaf K content decreases before shedding and the recovered K is deposited in the stem cortex and pith^18^. Japanese native poplar (*Populus maximowiczii*) also showed a decrease in leaf K concentration following dormant bud formation^19^. An increase in K^+^ concentration in xylem sap was observed during the winter season in field-grown *Populus nigra*^20^. These behaviors imply the existence of re-translocation mechanisms for K, and it is assumed that the potassium is transported to the organs that require it once it is resorbed. Potassium is transported through various systems within the plant body^21^. Epstein *et al*. showed that K^+^ absorption in barley roots is mediated by both a high-affinity- and a low-affinity biphasic transport process^22^. The high-affinity transport system (HATS) is up-regulated by a decrease in external K^+^ concentration. The low-affinity transport system (LATS), however, operates even when there is sufficient external K^+^ 23. Potassium ion uptake by the root symplast via HATS is mediated by the KUP/HAK/KT transporter family. There are thirteen such transporters in *Arabidopsis*^24^,^25^ and twenty-seven in rice^26^. Non-selective cation transport mechanisms such as voltage-independent cation channels (VICC) are categorized as LATS. In *Arabidopsis, AtCNGC* encodes VICC type channels. Twenty types of AtCNGC are present in the *Arabidopsis* genome.

Based on the above, it is assumed that the expression and function of K^+^ permeable transporters also regulate Cs^+^ translocation in various plant species and situations. Therefore, we investigated the relationship between the change in K localization induced by short day length and the behavior of Cs absorbed from the rhizosphere. To this end, ^137^Cs and ^42^K accumulations and gene expression patterns of major K^+^ transporters were analyzed using a model tree poplar, hybrid aspen T89.

## Results

### Amount of ^137^Cs in shoots was down-regulated under short-day conditions

Under a controlled growth cycle system in *Populus alba* L., the shift from long-day (LD) to short-day (SD) conditions decreased phosphate in the lower leaves^27^. This change suggests the existence of mechanisms for the re-translocation of phosphate from older- to younger (upper) leaves in response to with seasonal changes. Furukawa *et al*. indicated Ca^2+^ transport from root to shoot in *Populus maximowiczii* is also regulated by the shift from LD to SD^19^. Based on these facts, the uptake of Cs^+^ within the root and its behavior within the plant body in LD and SD conditions were compared. To measure seasonal variations in Cs^+^ uptake, a ^137^Cs^+^ solution was added to the growth media under LD3, LD9, and SD6 conditions (see Methods).

**Figure 1:**
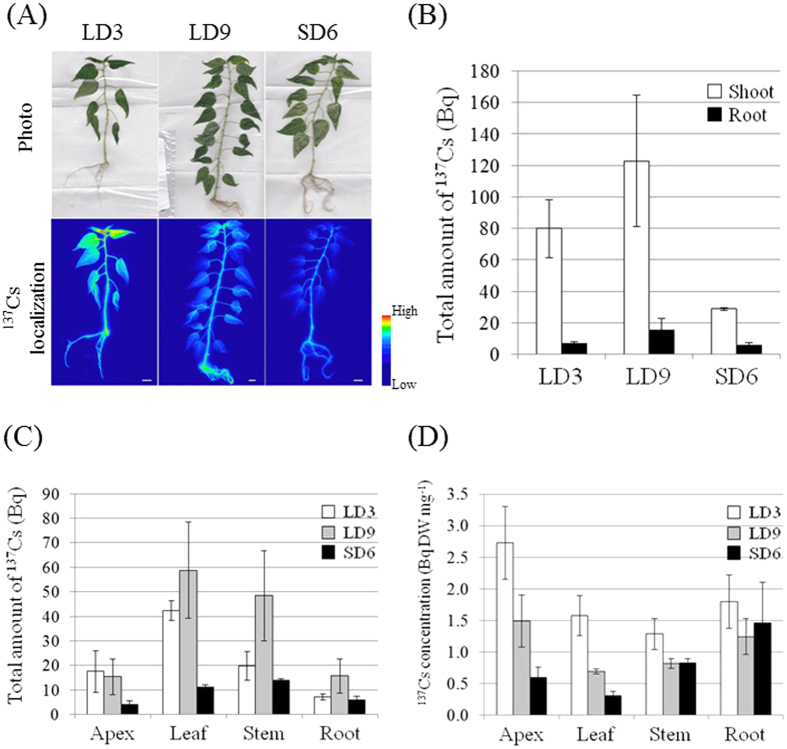
Effect of short-day transition on ^137^Cs uptake activity in poplar. (**A**) Localization of root-applied ^137^Cs under LD3, LD9, and SD6 conditions. The upper images are photos and the lower images are autoradiographs. Poplars were treated with ^137^Cs for 48 h. In the autoradiographs, color change from blue to red indicates ^137^Cs accumulation. Bar indicates 1 cm. (**B**) Alteration of the total amounts of ^137^Cs in poplar under transition to SD conditions. Poplars in each photoperiod were treated with ^137^Cs for 48 h. (**C**) Cesium-137 accumulations in each organ after 48 h treatment under LD3, LD9, and SD6 conditions. (**D**) Cesium-137 concentrations in each organ after 48 h treatment under LD3, LD9, and SD6 conditions. Three plants were tested for each photoperiod. Error bars indicate standard deviation.

Figure 1A shows the localization of ^137^Cs by root absorption under LD3, LD9, and SD6 conditions. In the LD3 plants, ^137^Cs was localized entirely and the radiation intensity around the apex was highest there. The LD9 plants were the same age as the SD6 plants and showed the same ^137^Cs behavior as the LD3 plants. In the SD6 plants, ^137^Cs was localized mainly in the stem and root and the total ^137^Cs was lower than that for the other plants. In LD3, LD9, and especially SD6 plants, all the nodes showed high amounts of ^137^Cs. The quantity of ^137^Cs in the shoots of SD6 plants was about 36.3% and 23.6% lower than that in the LD3 and LD9 shoots, respectively (Fig. 1B). On the other hand, the amount of ^137^Cs in the roots was similar for all three conditions. Cesium-137 accumulated mainly (48.8%) in the leaves under LD3 conditions (Fig. 1C). In LD9 conditions, ^137^Cs also accumulated to a large extent in the leaves (42.5%). Nevertheless, under SD6 conditions, the leaf ^137^Cs content was 32.1%, and organs containing the most ^137^Cs were the stems (39.7%). For the shoot apices, ^137^Cs levels were lower in SD6 plants than they were in LD3 and LD9, but the difference was not significant. The concentrations of ^137^Cs were highest in the apices of the LD3 and LD9 plants (Fig. 1D). Nevertheless, the decreases in the ^137^Cs concentrations in the apices and the leaves under SD6 conditions were significant, and the transition to SD suppressed Cs transport into the apices and the leaves.

### Potassium-42 uptake was constant under LD and SD conditions

**Figure 2:**
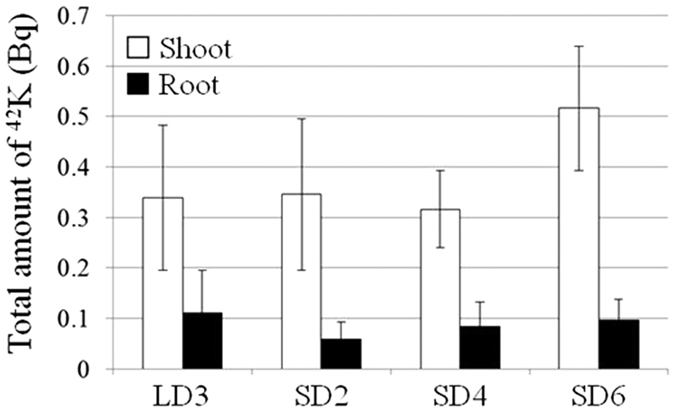
Effect of short-day transition on ^42^K uptake activity in poplar. Total amounts of ^42^K in poplar shoot and root under transition to SD. Plants under each photoperiod were treated with ^42^K for 24 h. Three plants were tested for each photoperiod. Error bars indicate standard deviation.

Based on the ^137^Cs uptake activity assays, it was expected that the amount of ^42^K absorbed through the root would also be down-regulated by the transition to SD. Poplar roots were treated with exogenous ^42^K and the amounts of ^42^K in the shoots and roots under LD3, SD2, SD4, and SD6 conditions were measured after 24 h incubation (Fig. 2). No difference was found in the amount of ^42^K in the roots among four conditions. The amount of ^42^K in the shoots at the early stage of SD was almost equivalent to that in the shoots at LD3. In contrast, the amount of ^42^K in the SD6 plant was slightly higher than it was in the other conditions, but the difference was not significant. These data suggest that the demand for K in the rhizosphere neither increased nor decreased by the transition to SD in the poplar for up to six weeks.

A comparison of Figs 1B and 2 indicates that ^137^Cs accumulation significantly decreased under SD6 condition, but ^42^K accumulation remained almost constant through the SD transition. This fact indicates that the decrease in Cs levels in the shoot is not explained by a simple reduction in transpiration rate under SD conditions and that important K^+^ uptake systems in poplar might be independently regulating Cs accumulations in it. It is also implied that the transporter responsible for Cs^+^ uptake in poplar might have only limited involvement in K^+^ uptake since no decrease in K accumulation was observed when Cs accumulation was low.

### *PttHAK-like1* was up-regulated by transition to short-day

We investigated under SD conditions the expression patterns of some candidate genes related to K^+^ transport. We focused on the KUP/HAK/KT family K^+^ transporters and a cyclic-nucleotide-gated channel (CNGC) type K^+^ channels. As for KUP/HAK/KT family genes, we concentrated on three genes in poplar. One of these was *Populus tremula* K^+^ *uptake transporter 1* or *PtKUP1* (Accession number, AJ299422; POPTR_0003s13370) which was identified in hybrid aspen^28^. *PtKUP1* was used in a complementation test with a K^+^ -uptake-deficient *E. coli* mutant. The addition of Cs^+^ to the culture media strongly inhibited the growth of *E. coli* expressing PtKUP1^28^. We also looked at two KUP/HAK/KT family transporters resembling AtHAK5. *Populus trichocarpa*, whose genome was elucidated in 2006^29^, has nine *AtHAK5* homolog genes in its genome, including *PtKUP1*. To evaluate the similarity between these putative K^+^ transporters, we constructed a phylogenetic tree of these genes and AtHAK5 homologs reported to be involved in K^+^ and Cs^+^ transport. We used their amino acid sequences (Fig. S1A). An *AtHAK5* homolog in barley, *HvHAK1*, also up-regulated its expression under K deficiency, and transgenic yeast expressing the *HvHAK1* gene showed an enhanced growth rate^30^. Like *HvHAK1*, rice *OsHAK5* also exhibits a high homology to *AtHAK5*^31^. The poplar gene POPTR_0010s10450 had the highest amino acid sequence homology with AtHAK5. POPTR_0001s00580 had the second highest. We identified POPTR_0010s10450 and POPTR_0001s00580 orthologues in the hybrid aspen T89 and named them *PttHAK-like1* and *PttHAK-like2*, respectively.

CNGC (cyclic-nucleotide-gated channel) may be a non-selective K^+^ channel which mediates K^+^ uptake by the root symplast^21^. In *Arabidopsis,* the CNGC channel AtCNGC2 shows K^+^ permeability^32^. A quantitative trait locus analysis indicated that *AtCNGC1* is associated with shoot K and Cs concentrations in *Arabidopsis*^12^,^33^. In *P. trichocarpa*, nine genes were selected as *AtCNGC1* homologs based on their amino acid sequences (see Supplementary Fig. S1B). Proteome BLAST analysis showed that POPTR_0012s01690 and POPTR_0015s02090 scored significantly higher than did others and the orthologues in hybrid aspen T89 were named as *PttCNGC1-like1* and *PttCNGC1-like2*, respectively.

**Figure 3:**
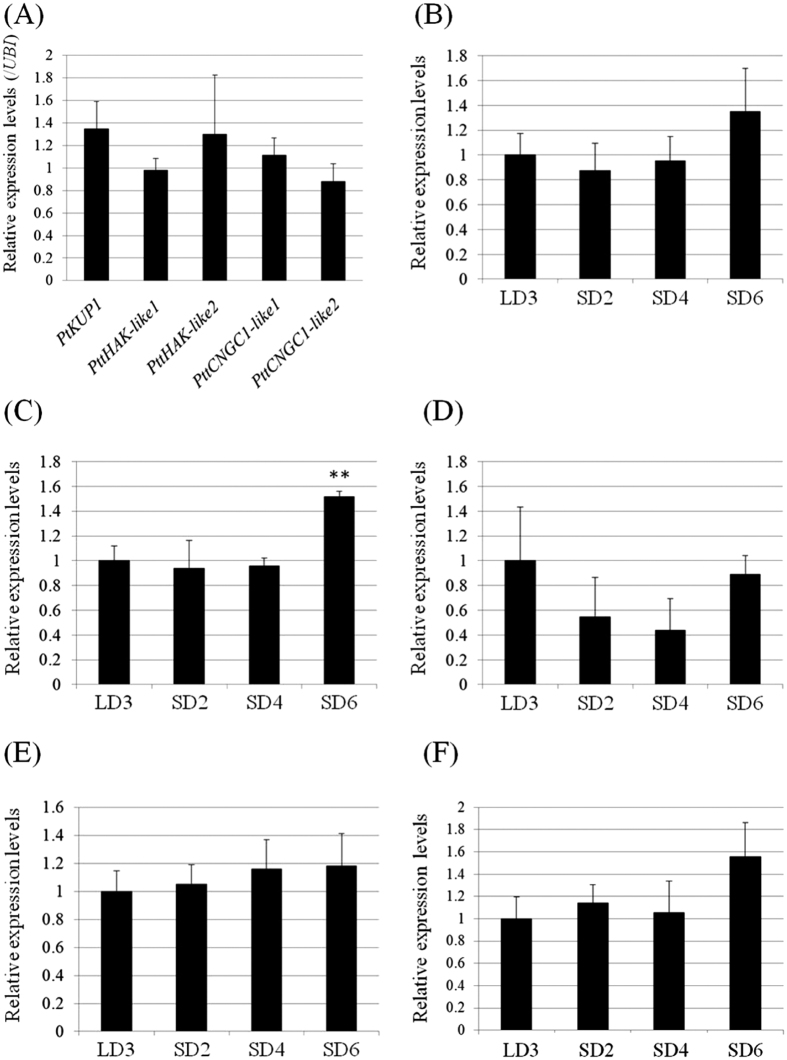
Effect of transition to SD on the transcriptional expression of the *KUP/HAK/KT* and *CNGC1* homolog genes in poplar root. Total RNA was isolated from the root and gene transcript levels were analyzed using qRT-PCR. *UBIQUTIN* was used as a reference gene. (**A**) Comparison of poplar K^+^ transport related genes under normal growth conditions (LD3). Three plants were tested for this analysis. Error bars indicate standard deviation. (**B**–**F**) Change in the expression levels of K^+^ transport related genes under transition to SD. (**B**) *PtKUP1*, (**C**) *PttHAK-like1*, (**D**) *PttHAK-like2,* (**E**) *PttCNGC1-like1*, and (**F**) *PttCNGC1-like2*. All gene expression levels were normalized by that for LD3. Error bars indicate standard deviation. * Indicates significant difference from the LD3 expression level (**<0.01). All primers used are listed in Table 1.

To determine which K^+^ uptake related gene is the most abundantly expressed among these five transporters, we evaluated the expression level of each gene under LD3 conditions. In poplar roots, no obvious differences were found in the expression levels of the genes selected (Fig. 3A). There may be redundancy in the expression of these K^+^ influx transporters under LD3 conditions. During the transition to the SD conditions, the expression of *PtKUP1* did not significantly change (Fig. 3B). *PttHAK-like1* showed steady expression until the transition to SD4 conditions and was up-regulated by about 1.5-fold under SD6 conditions (Fig. 3C). *PttHAK-like2* expression tended to decrease in SD2 and SD4 plants but maintained statistically steady-state transcription levels through SD transition (Fig. 3D). The expressions of *PttCNGC1-like1, PttCNGC1-like2* were also relatively constant under SD conditions (Fig. 3E,F).

There was a small but statistically significant increase in the expression level of *PttHAK-like1* under SD6 conditions but the amount of K absorbed through the root did not change with the transition to SD (Figs 2 and 3C). This inconsistency may be accounted for by the low elevation level of *PttHAK-like1* expression and the significant differences in the amino acid sequence of the poplar genes. To confirm, we sequenced the entire *PttHAK-like1* gene from the hybrid aspen T89. AtHAK5 and POPTR_0010s10450 in *P. trichocarpa* and PttHAK-like1 in hybrid aspen T89 and their alignment are shown in Supplementary Fig. S2. The AtHAK5 amino acid sequence showed 44.5% homology to POPTR_0010s10450 and 44.2% to PttHAK-like1. For the poplar, PttHAK-like1 and POPTR_0010s10450 shared 97.8% homology. The GEGGTFALY domain (AtHAK5-type transporters) is important for K^+^ and Cs^+^ selectivity^34^. Of these three genes, the GEGGTFALY domain was completely conserved and, consequently, there is no obvious explanation for the functional divergence.

Despite the steady ^42^K uptake manner through seasonal transitions, Cs accumulation activity was down-regulated under SD6 conditions. Therefore, the Cs^+^ and K^+^ transport systems are probably separately regulated in poplar.

## Discussion

Potassium is one of the most abundant essential plant nutrients. It is required for metabolism, photosynthesis, the tricarboxylic acid (TCA) cycle, glycolysis, and amino acid biosynthesis^35^. Maintaining enough K within the plant body is therefore quite important. For example, in K-deficient sunflowers, the carbon flux into the TCA cycle decreased due to changes in carbon distribution^36^. Potassium deficiency also inhibited sugar translocation in several plants^37^. Thus, the amount of K is closely tied to processes that maintain homeostasis in plants such as charge balance, pH regulation, and osmotic potential^35^. Potassium is the dominant solute in the xylem- and phloem saps of *Ricinus communis* and the circulation of K is required for plant growth and development^38^.

In this study, the relative amounts of ^42^K accumulated were compared over seasonal transitions. It was found that ^42^K accumulation remained constant until SD6. Dormant buds formed up to four weeks after the onset of the short-day treatment (data not shown); therefore, K re-translocation should have already started at SD6. Nevertheless, the results showed that ^42^K accumulation from root uptake and the expression of genes related to the root K^+^ uptake were almost constant (Figs 2 and 3B–F). It has been reported that the induction of AtHAK5 was enhanced by K^+^ deficiency^7^,^8^ or by Cs^+^ applications when there was sufficient K^+^ 39. Therefore, the slight increase in *PttHAK-like1* expression under SD6 might be a response to K^+^ starvation during the long growth period.

Despite the constant K accumulation pattern under SD conditions, Cs accumulation drastically decreased in SD6 plants (Fig. 1A,B). Cesium ion uptake and translocation are considered to be regulated by the plant K^+^ transport system but no down-regulation in the genes related to K^+^ uptake was identified during SD transition (Fig. 3B–F). It is known that plant mineral uptake mechanisms are regulated by protein activity level as well as gene expression^6^. Further nutrient transport activity analysis is necessary but these results suggest the possible existence of a novel uptake transporter which carries Cs^+^ much more efficiently than it does K^+^.

Since a decrease in Cs was observed only in the shoot (Fig. 1B), attention should be given to the transporters involved in Cs^+^ transfer between the root cells adjacent to the xylem and the xylem vessels themselves. Potassium-42 translocation from the root to the shoot was not affected by the transition to SD (Fig. 2). Therefore, K^+^ xylem loading might not be down-regulated, and there could be a Cs^+^ re-uptake pathway from the xylem sap to the root cells. This type of regulation was hypothesized for the Zn^2+^ transport system adjacent to root xylem vessels, and it may serve to keep shoot Zn^2+^ concentrations below toxic levels^40^. This mechanism would only be plausible if Cs^+^ specific transporters exist near the root xylem vessels—and these have not yet been found.

We did not find the K^+^ uptake transporter which was obviously up- or down regulated in the transition to SD. We used *Arabidopsis* eFP Browser (http://bar.utoronto.ca/efp/cgi-bin/efpWeb.cgi), which provides detailed information about the *Arabidopsis* gene expression site and various gene induction factors such as organic- and inorganic stressors. Expressions of the homologous *Arabidopsis* genes *AtKUP1, AtHAK5,* and *AtCNGC1* are not changed by short-day treatment or by exogenous abscisic acid. These expression patterns are consistent with our results and imply that short day length-induced regulation of K^+^ uptake is also unnecessary in poplar.

Unlike K accumulation, Cs accumulation did not remain constant, but drastically changed by day length transition. Therefore, the Cs^+^ uptake and translocation mechanisms differ from those for K^+^ in poplar. It was not determined why Cs accumulation was down-regulated but K accumulation was constant under the transition to SD conditions. It is clear, however, that Cs accumulation was affected by photoperiod.

## Methods

### Plant material and growth conditions

Hybrid aspen T89 (*Populus tremula* x *tremuloides*) (kindly provided by Prof, B. Sundberg, Swedish University of Agricultural Sciences, Sweden) were cultured in sterile pots in half-strength Murashige & Skoog (MS) medium under light- and temperature-controlled conditions (light 16 h, darkness 8 h, 23 °C; light intensity 37.5 μmol m^−1^ s^−1^). Each month, all plants were cut about five centimeters below the shoot apex and replanted in fresh MS medium.

### Measurement of ^137^Cs and ^42^K distributions in poplar

Poplars were grown under long-day (LD) conditions for three- and nine weeks in light- and temperature-controlled conditions (LD3 and LD9). Long-day conditions were as follows: light-period 16 h (light intensity 37.5 μmol m^−1^ s^−1^), dark-period 8 h, temperature 23 °C. To investigate the effects of seasonal transitions, the culture conditions were shifted to short-day (SD) for an additional two, four, and six weeks (SD2, SD4, and SD6) after the end of LD3 cultivation. Short-day conditions were as follows: light-period 8 h (light intensity 37.5 μmol m^−1^ s^−1^), dark-period 16 h, temperature 23 °C. ^137^CsCl (25 kBq, with 0.1 μM ^133^CsCl) or ^42^K (8 kBq, with 0.1 μM ^39^KCl) solutions were then added to the growth media to trace root absorption. The ^42^KCl solution was prepared using an ^42^Ar^+^-^42^K^+^ generator^41^,^42^. The purity of the ^42^K^+^ was verified from the gamma-ray spectra emitted by the test solutions using a germanium detector (GEM-type, ORTEC, USA). The decay of the ^42^K^+^ spectral peak (1525 keV) was monitored for 7 d as described in Kobayashi *et al*.^43^. The half-lives of the test solutions were measured with a liquid scintillation counter (LSC-6100, Hitachi Aloka Medical, Japan) and were theoretically identical to the actual half-life of ^42^K. Plants grown under LD and SD conditions were incubated with radioisotopes under the same photoperiods. Incubation times were 48 h and 24 h for the ^137^Cs and ^42^K experiments, respectively. Shoots and roots were separated and dried for 3 d at 50 °C. In the ^137^Cs assay using SD6 plants, plants were cut into four parts: apex (shoot apex and top three leaves), leaf (remaining leaves and petioles), stem, and root. To measure ^137^Cs and ^42^K radioactivity, the gamma counters AccuFLEX γ7001 (Hitachi Aloka Medical, Japan) and ARC-300 (Hitachi Aloka Medical, Japan) were used, respectively. The details of handling and measuring ^137^Cs and ^42^K were described in Kobayashi *et al*.^43^. Cesium-137 distribution was also investigated autoradiographically with a laser imaging scanner (FLA-9500, GE Healthcare, UK) in LD3, LD9, and SD6 plants. Significant differences between ^42^K and ^137^Cs quantities for each organ type and under each photoperiod were evaluated using one-way ANOVA.

### Acquisition of K influx transporter homologous gene nucleotide sequences

Full-length *AtHAK5* (At4G13420) and *AtCNGC1* (At5G53130) coding sequences were obtained from the *Arabidopsis* sequence database (TAIR; https://www.arabidopsis.org/). Eight homologous *HAK5* genes and nine homologous *CNGC1* genes were identified in poplar from the plant genomic resource (Phytozome; https://phytozom.jgi.doe.gov/pz/portal.html). *OsHAK1* (Os04g0401700) and *OsHAK5* (Os01g0930400) coding sequences were identified from RAP-DB, (http://rapdb.dna.affrc.go.jp/). The *HvHAK1* (Accession number: AF025292) coding sequence was searched using nucleotide BLAST in NCBI (http://www.ncbi.nlm.nih.gov/).

### Constructing the phylogenetic tree

The full-length coding sequences for the *Populus HAK5* and *CNGC1* homologs were converted to amino acid sequences, and then phylogenetic trees were created using the Maximum Likelihood method in MEGA application (Molecular Evolutionary Genetics Analysis, *ver.* 5.05).

### Gene expression analysis

Plant roots were flash-frozen in liquid nitrogen then pulverized using a mixer mill (QIAGEN, Germany). Total RNA was extracted using RNeasy Plant Mini Kit (QIAGEN). Quantitative real-time reverse-transcription PCR (qRT-PCR) was performed using One Step SYBR PrimeScript RT-PCR Kit ІІ (Takara, Japan) and 7300 Real Time PCR System (Applied Biosystems, USA). Three biological replicates were run for each photoperiod. An ubiquitin gene (Accession number: AF240445) was used as a reference gene in hybrid aspen T89. The *Ubiqutin* primers for qRT-PCR were the following: UBIQUTIN-F (5′-TGAACCAAATGATACCATTGATAG-3′) and UBIQUTIN-R (5′-GTAGTCGCGAGCTGTCTTG-3′). The gene expression analysis primers for *PtKUP1, PttHAK-like1, PttHAK-like2, PttCNGC1-like1,* and *PttCNGC1-like2* are listed in Table 1. Significant differences between each gene expression level were confirmed by one-way ANOVA.

### Cloning of the *PttHAK-like1* coding sequence

The full-length coding sequence of *PttHAK-like1* was cloned. The whole root of Hybrid aspen T89 grown until SD6 was harvested and stored at −80 °C. Total RNA was extracted using RNeasy Plant Mini Kit, and cDNA was synthesized from the extracted RNA with a ReverTra Ace (TOYOBO, Japan). The primers used for cloning *PttHAK-like1* were the following: *PttHAK-like1*: (5′-ATGGAAGGAGATGATGATCG-3′) and (5′-TTAGACCATGTATGTCATCCC-3′). The full-length coding sequence was amplified by PrimeSTAR GXL DNA Polymerase (Takara, Japan) and purified by Wizard SV Gel and PCR Clean-Up System (Promega, USA). *PttHAK-like1* was inserted into a pGEM T-Easy cloning vector by TA cloning (Promega, USA). The nucleotide sequences of *PttHAK-like1* were determined using a DNA sequencer (3130 Genetic Analyzer; Applied Biosystems, USA) and a Big-Dye terminator v3.1 sequencing standard Kit (Applied Biosystems, USA). The sequence was analyzed with Finch TV and BioEdit.

## Additional Information

**How to cite this article**: Noda, Y. *et al*. Short day length-induced decrease of cesium uptake without altering potassium uptake manner in poplar. *Sci. Rep.* **6**, 38360; doi: 10.1038/srep38360 (2016).

**Publisher's note:** Springer Nature remains neutral with regard to jurisdictional claims in published maps and institutional affiliations.

## Supplementary Material

Supplementary Figures

## Figures and Tables

**Table 1:** Specific primers of K^+^ influx genes in poplar for qRT-PCR.

Gene	Gene Locus	Primer (5′–3′)
*PtKUP1*	POPTR_0003s13370	AGAGCTCTTGGAAGCAAAAG
		ACACTAGGGGACCGACAGTT
*PttHAK-like1*	POPTR_0010s10450	CTTATTTACTGGCTCACGGG
		TATTAGCAGCACCGGCTCTA
*PttHAK-like2*	POPTR_0001s00580	TTGTGCACATACTGGGGAAT
		AGCTGCACTGTTCTGTCTGC
*PttCNGC1-like1*	POPTR_0012s01690	CGGCAAGAGGAAAATAGATT
		TCGTAGAGCACGCAAAATGT
*PttCNGC1-like2*	POPTR_0015s02090	CACCAAGTTTAGGTGCCACA
		GCAGCATTGGTGGAACTCTA
