# Supplementary material for: Short day length-induced decrease of cesium uptake without altering potassium uptake manner in poplar
Source: Sci Rep. 2016 Dec 7;6:38360. doi: 10.1038/srep38360 (PMC5141437; doi:10.1038/srep38360)
Supplement: Supplementary Figures [file srep38360-s1.doc]

**Supplementary information**

**Short day length-induced decrease of cesium uptake without altering potassium uptake manner in poplar**

Yusaku Noda^1^, Jun Furukawa^2,3^, Tsutomu Aohara^2^, Naoto Nihei^4^, Atsushi Hirose^4^, Keitaro Tanoi^4^, Tomoko M. Nakanishi^4^ and Shinobu Satoh^2^

^1^ Graduate school of Life and Environmental Sciences, University of Tsukuba, Tsukuba 305-8572, Japan. ^2^ Faculty of Life and Environmental Sciences, University of Tsukuba, Tsukuba 305-8572, Japan. ^3^ Center for Research in Isotopes and Environmental Dynamics, University of Tsukuba, Tsukuba 305-8577, Japan. ^4^ Graduate school of Agricultural and Life Sciences, The University of Tokyo, Tokyo 113-0032, Japan

**Supplementary Figure S1. Phylogenetic trees of poplar KUP/HAK/KT transporters.** Phylogenetic trees were constructed using Maximum Likelihood method with MEGA phylogeny application. Locus tag; AtHAK5, At4g13420; AtCNGC1, At5g53130; OsHAK1, Os04g0401700; OsHAK5, Os01g0930400. HvHAK1 Genebank accession number, AF025292. (A) Poplar AtHAK5 homologous genes with several plants orthologues. (B) AtCNGC1 homologous genes in poplar.

**Supplementary Figure S2. Amino acid alignment with AtHAK5, POPTR_0010s10450, and PttHAK-like1.** Amino acids were aligned by ClustalW program. Black enclosure means homologous region in three HAK amino acid sequences. Black bars indicate transmembrane domain of PttHAK-like1. Red box indicates GEGGTFALY domain. X represents termination codon.

**Supplementary Figure S1**

**
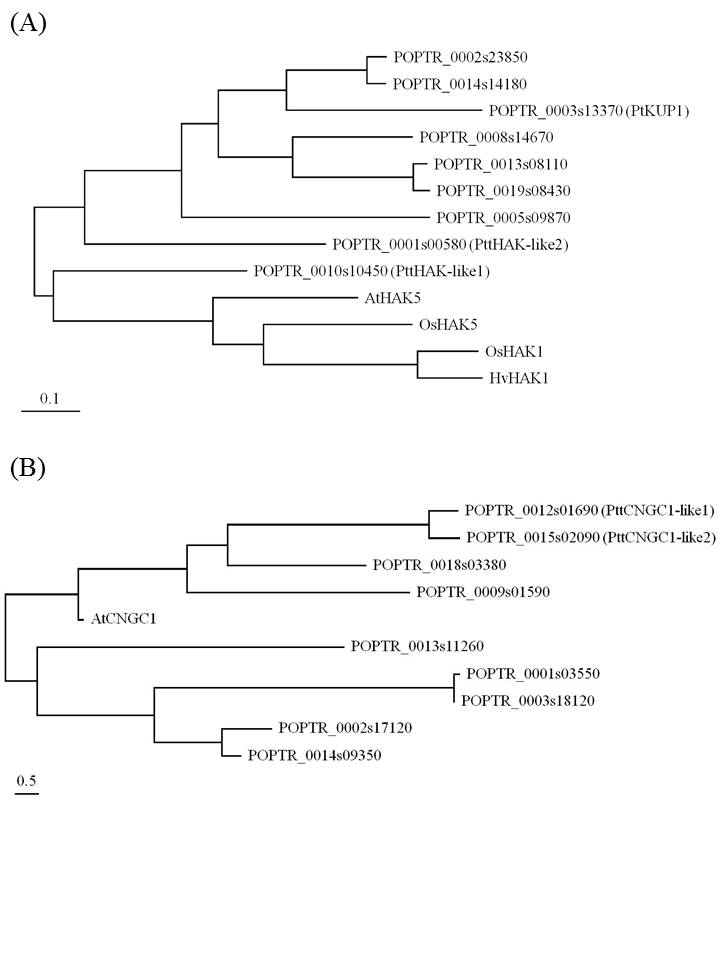
**

**Supplementary Figure S2**

**
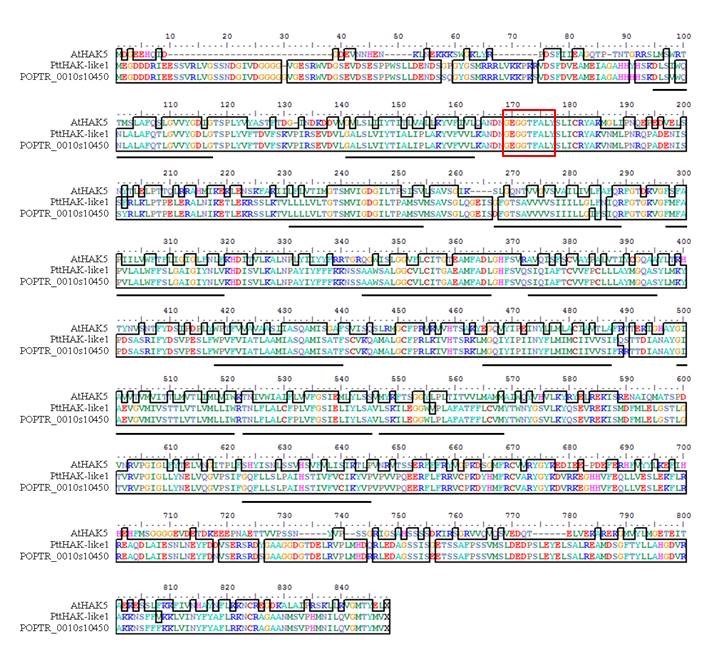
**
